# Self-efficacy and well-being in the association between caregiver burden and sleep quality among caregivers of elderly patients having multiple chronic conditions in rural China: a serial multiple mediation analysis

**DOI:** 10.1186/s12912-023-01587-0

**Published:** 2023-11-13

**Authors:** Ziyue Yang, Fengye Sun, Lingrui Zhao, Tingwei Hu, Xin Lin, Yufang Guo

**Affiliations:** School of Nursing and Rehabilitation, Shandong University, 44 Wen Hua Xi Road, Jinan, 250012 Shandong China

**Keywords:** Multiple chronic conditions, Sleep quality, Caregiver Burden, Self Efficacy, Well-being, China, Rural Population

## Abstract

**Background:**

Caregivers of elderly patients with multiple chronic conditions have heavy caregiver burden and poor sleep quality, which has an important impact on both caregivers and patients. This study aimed to examine among rural caregivers of elderly patients who have multiple chronic conditions in China, whether self-efficacy and well-being mediate the link between caregiver burden and sleep quality.

**Methods:**

The study recruited 325 caregivers of elderly patients having multiple chronic conditions in rural China. Several measures including the Caregiver Burden Inventory (CBI), Athens Insomnia Scale (AIS), General Self-Efficacy Scale (GSES) and Index of Well-Being (IWB) were utilized to collect data. Structural equation modeling was employed to study the relationships among caregiver burden, sleep quality, self-efficacy, as well as well-being.

**Results:**

Significant correlations were found between the measured variables (each *p* < 0.01). Self-efficacy and well-being acted as mediators in the link between caregiver burden and sleep quality, accounting for 10.94% and 15.63% of the total effect, respectively. In addition, self-efficacy and well-being had a serial multiple mediating effect in the association between caregiver burden and sleep quality, with this mediating pathway, explaining 9.93% of the total effect.

**Conclusions:**

Caregivers of elderly patients having multiple chronic conditions in rural China experienced poor sleep quality due to the caregiver burden. Self-efficacy and well-being had serial mediating roles on the relationship between caregiver burden and sleep quality. Effective interventions should be developed to improve self-efficacy and well-being of caregivers, reduce their caregiver burden and, eventually, improve their sleep quality.

## Background

The worldwide population is experiencing rapid aging, and China is no exception [1]. By 2050, the number of elderly is expected to be over 1.5 billion [2]. China’s aging population has been steadily increasing, leading to the country being classified as an aging society since 2000, and the proportion of individuals over the age of 65 continues to rise, reaching 13.50% in 2020, up from 8.87% in 2010 [3]. The shift in demographics is more prominent in rural areas of China, where the aging population is more severe compared to urban areas [4]. The elderly have a high incidence of chronic diseases [5], and multiple chronic conditions (MCC) [6], which means the cooccurrence of two or more chronic conditions by definition [7]. Data showed that 86.67% of elderly people suffer from one or more chronic diseases, and 65.16% of elderly individuals with chronic diseases in China suffer from MCC [8]. These factors result in a disproportionate health and cost burden [9], affecting both patients and caregivers significantly [10, 11].

Caregiver burden refers to the caregivers multifaceted strain level perceived in caring their family members or loved ones over time [12]. Studies have found that informal caregivers commonly provide care for older adults with MCC, and consequently, they often experienced caregiver burden [13]. Caregivers may feel overwhelmed by the demands of care, with many reporting negative physical and mental effects. In one study, 53.40% of caregivers reported that they needed to provide whole-day care for older adults, and 57.70% reported that they suffered negative physical and mental effects because of caring for patients without taking care of their own health [14]. Research shows that in China, the caregiver burden for elderly patients having chronic diseases is moderate to high [15]. Caregiver burden can significantly affect the caregivers’ mental and physical health, social relationships, and overall health [16–21].

The stress-coping theory, proposed by Lazarus and Folkman [22], proposes a process by which a person copes with a stressful condition associated with physical and mental discomfort in order to produce appropriate and effective adaptations in behavior. Two continual stages, which was named as the cognitive appraisals and the coping efforts, constitute the coping process. During the former, individuals assess the potential consequences of specific environmental events for themselves, including potential threats and challenges. The term “coping efforts” means the ongoing cognitive and behavioral strategies employed by ones in managing stressors that are perceived as exceeding their personal resources. According to stress coping theory, individuals who experience high levels of caregiver burden may develop a decreased sense of self-efficacy, which can lead to poorer physical and mental health, as well as their worse sleep quality [23]. Research consistently demonstrates a strong correlation between caregiver burden and low sleep quality. Caregivers experiencing higher levels of burden were more possible to have worse sleep quality [24, 25]. Poor sleep quality result in negative affective, behavioral, and cognitive responses. This cycle of sleep problems can perpetuate itself, negatively impacting caregivers’ health-related life quality. Therefore, it is crucial to explore the influencing pathway of caregiver burden on sleep quality. According to the stress coping theory, this study regards the caregiver burden as a stressful condition and explores the internal mechanism of its influence on the sleep quality .

Self-efficacy is the belief that a person has in their own capacity to complete a task or accomplish a specific goal under certain circumstances [26]. According to stress coping theory, individuals who experience high levels of caregiver burden may develop a decreased sense of self-efficacy, which can lead to poorer physical and mental health, as well as their worse sleep quality. This is because the constant demands of caregivers can create a feeling of being overwhelmed, leading to a perceived lack of control and a sense of helplessness [27]. Study has shown self-efficacy can influence sleep quality by affecting individuals’ sleep-related behaviors [28]. Numerous studies have shown the significant correlation between caregiver burden and self-efficacy, as well as the significant correlation between caregiver’s self-efficacy and sleep quality [29, 30]. However, the specific pathways linking these variables are still not well understood. Hence, it is possible self-efficacy had a mediation effect between caregiver burden and sleep quality.

Raz defines well-being as an unwrapping of the notion of a person experiencing good life [31]. Research has shown a significant link between well-being and caregiver burden, suggesting that lower levels of caregiver burden could be related to greater well-being [32]. Some studies also suggest that well-being can predict sleep quality [33]. Similar to self-efficacy, according to stress coping theory, individuals who experience high levels of caregiver burden may develop a decreased well-being, which can lead to worse sleep quality. Despite evidence exhibited the associations among self-efficacy, caregiver burden and sleep quality, the specific mechanisms underlying these associations remain unclear. In light of the results presented, it is hypothesized that well-being may have a mediation effect between caregiver burden and sleep quality.

**Fig. 1:**
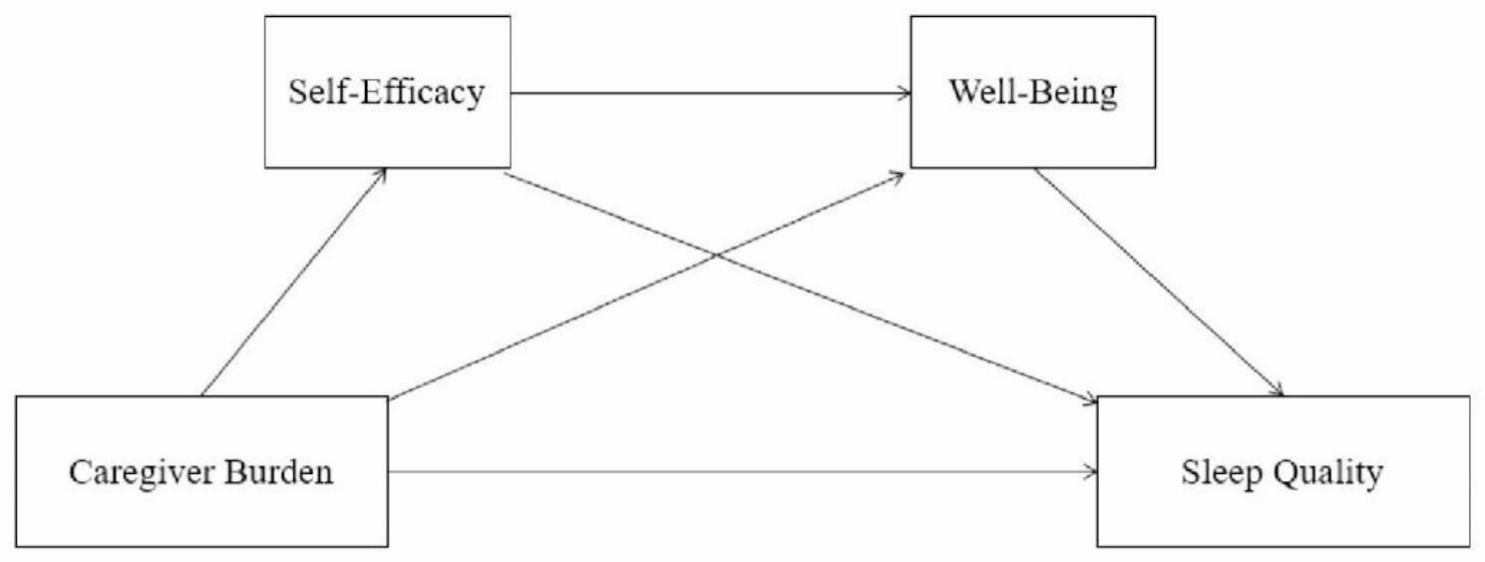
Hypothesized mediation model

In summary, this study adapts the stress-coping theory [34] to propose that self-efficacy and well-being play a role in the process whereby caring burden, a stressful event, affects sleep quality. Meanwhile, previous research has shown that self-efficacy is significantly linked to the well-being [35]. Therefore, this study inferred that improving self-efficacy may have a potential effect on caregivers’ emotional well-being, which may lead to improved sleep quality. In summary, the stress coping theory offers a theoretical framework for comprehending the sequential mediation effects of caregiver burden, self-efficacy, well-being, and sleep quality. Figure 1 illustrate this study’s theoretical framework.

This research aimed to examine the association between caregiver burden and sleep quality, as well as explore the potential mediating roles of self-efficacy and well-being on the link between caregiver burden and sleep quality among the caregivers providing care to MCC patients residing in rural China.

## Methods

### Aims

This study explored whether self-efficacy and well-being mediate the link between caregiver burden and sleep quality.

### Design

This is a cross-sectional design study.

### Participants

In the study, a questionnaire survey was conducted among caregivers from rural areas of the Xinjiang Uygur Autonomous Region, Hebei, Shandong, Sichuan, Shanxi, Heilongjiang, Jiangsu, Hubei, Gansu, Anhui, Henan, Zhejiang and Shaanxi provinces in China using convenience sampling. Based on the following criteria, participants were selected : (1) patients and caregivers lived in rural areas for one or more years; (2) patients were aged 60 years and above; (3) patients had at least two chronic diseases, such as hypertension, coronary heart disease, cerebral infarction, diabetes, chronic obstructive pulmonary disease, and osteoarthritis; (4) the caregiver had a certain blood or geographical association with the patient and was responsible for the daily life care; (5) caregivers were not paid; and (6) the duration of care was more than 3 months. Participants who had a history of alcohol and drug dependence, a history of mental illness, or did not wish to take part in the study were excluded from the research. This research’s sample size was determined according to the requirement that the minimum sample size was 5–10 times the number of variables [36]. A total of 11 dimensions of statistical analysis were included in this study (1 item from the general situation questionnaire, 5 items from the Caregiver Burden Inventory, 1 item from the General Self Efficacy Scale, 2 items from the Index of Well-Being, and 2 item from the Athens Insomnia Scale). This study determined that a minimum of 110 cases were needed, with an additional 10% added to account for potential sample loss or non-cooperation, bringing the final sample size to 121.

### Measurements

#### Demographic and clinical characteristics form

This study assessed demographic variables include gender, age, marital status, educational level, religious belief, lifestyle, number of chronic diseases diagnosed by doctors and medications of the patients. Additionally, the caregiver’s identity to the patient, caregivers’ age, gender, marital status, monthly income per caregiver household as well as perceived health status were gathered.

#### Caregiver burden inventory (CBI)

The Caregiver Burden Inventory (CBI) was employed to evaluate caregiver burden, which is translated and revised by Yue Peng et al [37]. The CBI is a multidimensional scale that measures various aspects of caregiver burden related to take care of individuals, consisting of 24 items. The inventory is evaluated with a 5 point Likert-type scale and is divided into 5 dimensions, whose items ranged from zero (not at all descriptive) to four (very descriptive). In this scale, higher score indicates greater burden, with a possible score range of 0 to 96. The Chinese version of the CBI’s reliability and validity have been showed according to prior research. In this study sample, the Cronbach’s alpha was 0.937, indicating high internal consistency and good reliability.

#### Athens insomnia scale (AIS)

The AIS was employed to examine caregivers’ sleep quality [38, 39]. The AIS is a validated scale comprising eight self-reported items used in clinical diagnosis and research, and in the Chinese population, it has been validated. Respondents rated the eight self-reported items employing a 4-point Likert scale, in which the options ranged from 0 (no problem) to 3 (very severe problem). As a result, all items’ overall score ranged from 0 to 24, as well as worse and more serious insomnia symptoms are indicated by higher scores. With Cronbach’s α of 0.89, previous studies have reported this scale’s high internal consistency. According to this research sample, the AIS’s Cronbach’s α was 0.855.

#### General self efficacy scale (GSES)

In this study, researchers utilized the General Self-Efficacy Scale’s Chinese version, which was translated and revised by Wang et al [40] to examine caregiver self-efficacy. With a four-point Likert scale, GSES was consisted of ten items and developed by Schwarzer et al. originally. It has a Cronbach’s α coefficient of 0.87, showing that it had good validity and reliability. In addition, the GSES is a self-reporting scale that assesses one’s perceived self-efficacy. The items ranged from 1 (completely wrong) to 4 (absolutely right). The GSES overall score is calculated by diving by 10 after adding up the scores of the 10 items. The greater score means greater self-efficacy. In this research sample, the GSES’s Cronbach’s α was 0.856.

#### The index of well-being (IWB)

As a validated tool for assessing well-being [41], the index of Well-Being Index consists of nine statements that evaluate a person’s current level of subjective well-being, which was developed by Campbell. Higher scores on the scale indicate better well-being, with each item ranging from 2.1 to 14.7. Previous research conducted with Chinese populations has reported the index of Well-being Chinese version had good reliability and validity. And according to this research sample, the Cronbach’s α of 0.941 for this scale has high internal consistency, meaning its items are measuring the same construct. This suggests that this scale is reliable and consistent in measuring caregivers’ well-being.

### Data collection

The study followed ethical guidelines and obtained approval from School of Nursing and Rehabilitation’s Ethics Committee, Shandong University. These participants completed self-rating questionnaires from August to September 2022 after being informed about the study and giving written consent. A total of 702 questionnaires were delivered and, among them, 485 fully met the inclusion and exclusion criteria, with a response rate of 69.09%. After excluding incomplete questionnaires with missing data, 325 questionnaires were included in the analysis.

### Ethical consideration

This study was approved by the college’s ethics committee (number 2022-R-125). All participants in the study are voluntary participation.

### Data analysis

SPSS 26.0 (IBM) was employed to analyze all data of this study. The study utilized a mediation model where caregiver burden was treated as the independent variable, sleep quality was the dependent variable, as well as the caregivers’ self-efficacy and well-being were mediating variables. Patient age and educational level, as control variables, were also included in the model to consider their potential impact in the associations among the measured variables. Descriptive statistics were calculated for each case. Common method bias refers to the bias caused by common method variance [42]. Two effective methods were employed in the research to control common method bias. These methods were the use of an anonymous form and individual items’ reverse scoring. Harman one-way test was further used and five factors that characteristic roots greater than 1 were identified [43]. The result showed that 26.63% of the variance can be explained by the largest common factor, which was lower than 40%. Therefore, no significant common method bias was found in the study. To determine the correlation between variables, Pearson’s r analysis was performed. To test the self-efficacy and well-being’s serial multiple mediation effect between caregiver burden and sleep quality, Model 6 from the macroprogram PROCESS was implemented. Bootstrapping, which involves drawing multiple random samples from the dataset, was utilized in the research to investigate the reliability of the statistical model. More specifically, for each regression coefficient, 5000 samples were drawn to compute the 95% confidence interval (CI). For each effect’s significance’s determination, the CI for each coefficient, ranging from Boot LLCI to Boot ULCI, was examined to ascertain whether it included zero or not. It was considered statistically significant when the effects with CIs that did not include zero.

## Results

### Descriptive analysis

The 325 participants’ demographic characteristics in this research were displayed by Table 1. The mean age for patients was 74.62 years. 48.0% were male and 52.0% were female. The caregivers’ mean age was 51.42 years, with 56.6% male and 43.4% female.

**Table 1:** Demographic characteristics (N = 325)

Variable	Category	Number(n)	Percentage(%)/Mean ± SD
Patient gender	Male	156	48.0%
	Female	169	52.0%
Patient age		325	74.62 ± 0.431
Patient lifestyle	Live alone	145	44.6%
	Live with children	112	34.5%
	Live with spouse	52	16.0%
	Others	16	4.9%
Number of chronic diseases of patients which has been diagnosed by doctors	2	200	61.5%
	3	98	30.2%
	4	22	6.8%
	5	4	1.2%
	6	1	0.3%
The patient’s medication status	Three times a day	119	36.6%
	Twice a day	126	38.8%
	Once a day	63	19.4%
	Once a week	6	1.8%
	Once a month	1	0.3%
	Others	10	3.1%
Caregiver’s identity	Spouse	65	20.0%
	Son	131	40.3%
	Daughter-in-law	43	13.2%
	Daughter	63	19.4%
	Others	23	7.1%
Caregiver age		325	51.42 ± 13.517
Caregiver gender	Male	184	56.6%
	Female	141	43.4%
Caregivers perceive health status	Very good	51	15.7%
	Better	145	44.6%
	Ordinary	118	36.3%
	Worse	11	3.4%

### Correlation between caregiver burden, self-efficacy, well-being and sleep quality

Table 2 presents this study’s findings of the Pearson correlation analysis, which demonstrated significant associations among caregiver burden, self-efficacy, well-being, as well as sleep quality (each *p* < 0.01). Specifically, caregiver burden showed significant correlations with self-efficacy, well-being, as well as sleep quality. Self-efficacy of the caregivers was significantly correlated with both well-being and sleep quality, whereas well-being was significantly correlated with sleep quality.

**Table 2:** Correlation between all variables

Variable	M	SD	Caregiver burden	Self-efficacy	Well-being	Sleep quality
Caregiver burden	33.6508	16.76749	1			
Self-efficacy	27.0742	4.04259	-0.228^**^	1		
Well-being	42.7095	11.62771	-0.247^**^	0.500^**^	1	
Sleep quality	5.0209	3.61413	0.411^**^	-0.474^**^	-0.583^**^	1

Note: **p < 0.01(two-tailed)

### Mediating effects of self-efficacy and well-being on the link between caregiver burden and sleep quality

**Fig. 2:**
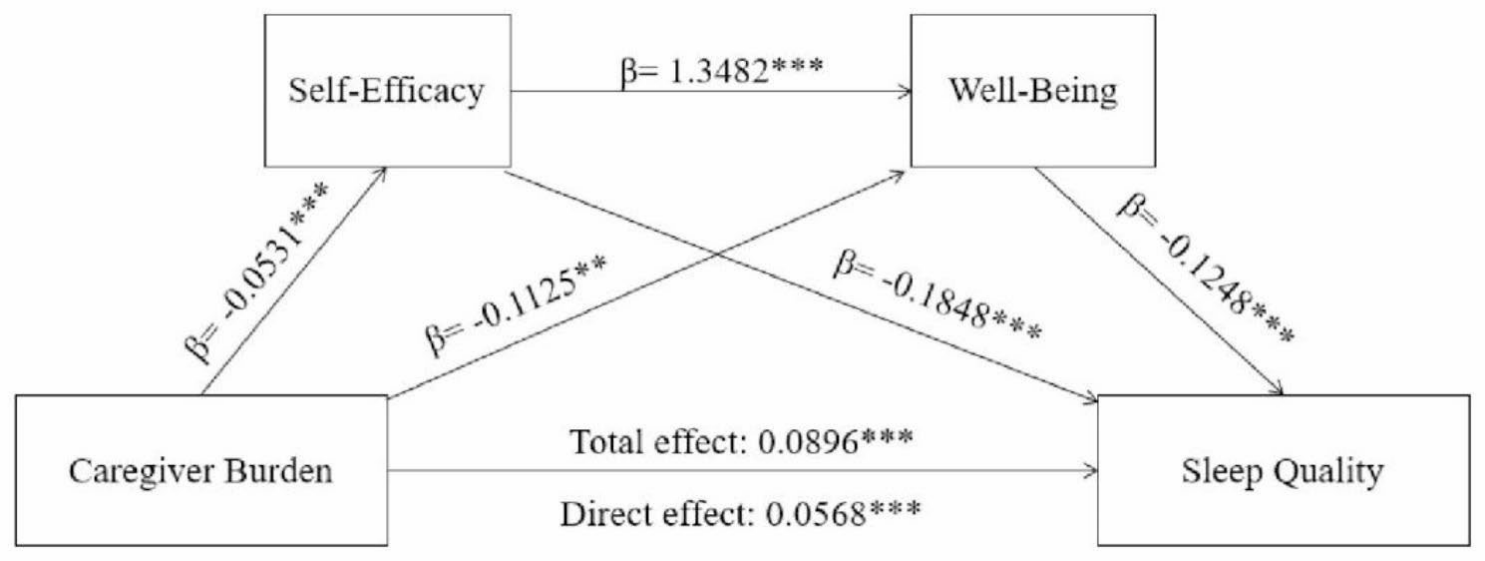
The chain mediation of self-efficacy and well-being between caregiver burden and sleep quality(**p < 0.01, ***p < 0.001) Note. **p < 0.01, ***p < 0.001

The mediating effects of self-efficacy and well-being are shown in Table 3; Fig. 2.

**Table 3:** The mediation analysis results

	Effect	BootSE	BootLLCI	BootULCI	Ratio of indirect to total effect	Ratio of indirect to direct effect
Total Effect	0.0896***	0.0110	0.0680	0.1112		
Direct Effect	0.0568***	0.0094	0.0384	0.0753		
Total Indirect Effect	0.0328***	0.0069	0.0195	0.0468	36.61%	57.75%
Ind1: Caregiver Burden→ Self-Efficacy→ Sleep Quality	0.0098***	0.0037	0.0036	0.0180	10.94%	17.25%
Ind2: Caregiver Burden→ Well-Being→ Sleep Quality	0.0140***	0.0047	0.0048	0.0234	15.63%	24.65%
Ind3: Caregiver Burden→ Self-Efficacy→ Well-Being→ Sleep Quality	0.0089***	0.0027	0.0040	0.0148	9.93%	15.67%

Note: *** p < 0.001

This paper shows the total indirect effect, which includes three different pathways, was statistically significant and accounted for 36.61% of the total effect, with a value of 0.0328 (95% CI: [0.0195, 0.0468], *p* < 0.001). The indirect effects were observed via self-efficacy, well-being, and serial mediation via self-efficacy and well-being. Caregiver Burden→Self-Efficacy→Sleep Quality, Caregiver Burden→Well-Being→Sleep Quality, and Caregiver Burden→Self-Efficacy→Well-Being→Sleep Quality explained 10.94% (95% CI: [0.0036, 0.0180], *p* < 0.001), 15.63% (95% CI: [0.0048, 0.0234], *p* < 0.001), and 9.93% (95% CI: [0.0040, 0.0148], *p* < 0.001) of the total effect, respectively. All of the 95% confidence intervals (CIs) did not overlap with zero, indicating that all indirect effects were statistically significant.

## Discussion

The study adds to the current body of literature which is related to the caregivers of elderly patients with MCC by examining the mechanism that underlies the link between caregiver burden and sleep quality. Moreover, the study align with prior study [44] demonstrating caregiver burden has an adverse influence on sleep quality. This study is also consistent with the conclusions of previous studies about the mediating effects of self-efficacy [45] and well-being [46] on the association between caregiver burden and sleep quality. Moreover, this study provides further insights into the underlying mechanism by showing that self-efficacy and well-being play serial multiple mediating roles in the association between caregiver burden and sleep quality. This study suggested that caregiver burden could reduce self-efficacy and well-being, which in turn leads to poorer sleep quality.

### Independent mediation of self-efficacy and well-being

This study has revealed that self-efficacy significantly mediated the link between caregiver burden and sleep quality. This finding highlights the essential influence of self-efficacy in the management for caregiver responsibilities, as well as the maintenance of health. Feng’s study suggests that high self-efficacy can lessen the adverse effects of burden on sleep quality [29]. According to stress coping theory, an individual’s perceived self-efficacy impacts their appraisal of stressful situations and ability to cope with stressors. Caregivers having greater self-efficacy tend to have a more positive outlook towards stressors and may approach them with a problem-solving attitude rather than feeling overwhelmed or helpless, which can decrease emotional distress and enhance active coping efforts when facing high levels of caregiver burden [47, 48]. These factors, in turn, may improve the caregiver’s ability to regulate their emotions, leading to better sleep quality [49]. By mediating the relationship, self-efficacy is a critical factor in how caregiving stress affects sleep quality. Specifically, self-efficacy is one of crucial factors in the caregiving stress process as it influences how caregivers perceive and cope with stressors.

In this study, the caregiver burden influenced sleep quality indirectly through well-being, and the mediating effect of well-being was 0.0140. Liu’s research shows that well-being can lessen the adverse effects of burden on sleep quality [46]. Stressful events (such as caregiver burden) affect sleep quality by reducing well-being, and increasing well-being not only directly help to improve sleep problems, but also buffer the effects of stress on sleep and indirectly promote sleep quality. Caregiver burden can result in negative emotional responses, including anxiety and depression, which may subsequently decrease the caregiver’s overall well-being [50]. Decreases in well-being can then lead to worse sleep quality because of the emotional and physical burden that caregivers experience. In summary, this study enhanced the significance of well-being as one of mediators between caregiver burden and sleep quality. Well-being appeared to play a crucial role in how caregivers perceive and cope with stressors, ultimately impacting their sleep quality.

### The serial mediating role of self-efficacy and well-being

The most interesting finding of the study was that self-efficacy and well-being acted as serial multiple mediators between caregiver burden and sleep quality. The stress coping theory suggests that caregiver burden can increase stress levels and negatively impact sleep quality. However, individuals having great self-efficacy are better equipped to manage caregiver responsibilities, as well as cope with stress, leading to increased well-being [51]. Higher levels of well-being can positively affect sleep quality, contributing to better physical and emotional health. Multiple studies have shown that self-efficacy [52] and well-being [53] play a positive role in many aspects of an individual’s physical and mental health. Therefore, individuals with high self-efficacy may experience higher levels of well-being and better sleep quality. The stress-coping model provides a framework for understanding how caring responsibilities, self-efficacy, well-being, and sleep quality are interrelated. Caregiving responsibilities can increase stress levels and reduce self-efficacy beliefs, leading to reduced well-being. However, individuals with high self-efficacy are better equipped to view stressors as challenges, leading to more effective stress management, higher levels of well-being, and ultimately better sleep quality. In summary, the findings indicate that in this link between caregiver burden and sleep quality, self-efficacy and well-being play crucial roles as serial mediators.

### Strengths and limitations

The research has the advantage of focusing on caregivers from rural areas in China, which is a population that has been underrepresented in previous studies on this topic. Additionally, this study has provided important insights into the mediating mechanisms that explain this link between caregiver burden and sleep quality. These findings could be useful in developing effective interventions for improvement of caregivers’ well-being’s.

Despite these significant findings, it is essential to acknowledge the limitations of this paper and these findings should be interpreted with caution. First, this research’s generalizability may be limited as the result of the convenience sampling method used. Second, causal inference cannot be made for the cross-sectional design of this research. Therefore, longitudinal studies are necessary to clarify this association discussed. Third, according to this study, self-reported measurements were used to record variables, which can lead to some differences and bias. Fourth, although this study identified a significant mediation effect of self-efficacy and well-being between caregiver burden and sleep quality, the magnitude of the mediating effect was relatively small. This study thinks that this may be limited by the low educational level of some caregivers during the survey. This study suggests that future research can find a questionnaire more suitable for the educational level of caregivers in rural areas when selecting questionnaires. Fifth, expanding the scope of variables in future studies may offer a more comprehensive and detailed comprehension of the links among caregiver burden, sleep quality, and additional relevant factors, such as social support, potentially shedding more light on effective interventions for caregivers of elderly patients having multiple chronic conditions residing in rural China.

## Conclusions

To summarize, this study explored examined the mediation effect of self-efficacy’s and well-being between caregiver burden and sleep quality of caregivers providing care to MCC patients residing in rural China. The study indicates that reducing caregiver burden, enhancing self-efficacy and well-being can effectively improve sleep quality. Effective interventions aimed at enhancing the self-efficacy and well-being of caregivers should be implemented to improve their sleep quality. Furthermore, supportive policies and care knowledge trainings to reduce caregiver burden for family caregivers need to be developed for improving their sleep quality, which could promote their health and quality of care. Specific measures can include that township health center, other grass-roots health and medical institutions in rural areas regularly monitor the levels of caregivers’ self-efficacy and well-being, and implement targeted interventions to promote self-efficacy and well-being according to research guidance. These will be conducive to improving the physical and mental health of both caregivers and patients, thus contributing to the construction and development of healthy aging.

## Data Availability

The datasets used and/or analysed during the current study are available from the corresponding author on reasonable request.
